# Tumor-associated macrophages: orchestrators of cholangiocarcinoma progression

**DOI:** 10.3389/fimmu.2024.1451474

**Published:** 2024-09-03

**Authors:** Fei Chen, Jian Sheng, Xiaoping Li, Zhaofeng Gao, Lingyu Hu, Minjie Chen, Jianguo Fei, Zhengwei Song

**Affiliations:** ^1^ Department of Surgery, The Second Affiliated Hospital of Jiaxing University, Jiaxing, China; ^2^ Department of Research and Teaching, The Second Affiliated Hospital of Jiaxing University, Jiaxing, China

**Keywords:** CCA, TAMs, macrophage reprogramming, immune evasion, tumor microenvironment

## Abstract

Cholangiocarcinoma (CCA) is a rare but highly invasive cancer, with its incidence rising in recent years. Currently, surgery remains the most definitive therapeutic option for CCA. However, similar to other malignancies, most CCA patients are not eligible for surgical intervention at the time of diagnosis. The chemotherapeutic regimen of gemcitabine combined with cisplatin is the standard treatment for advanced CCA, but its effectiveness is often hampered by therapeutic resistance. Recent research highlights the remarkable plasticity of tumor-associated macrophages (TAMs) within the tumor microenvironment (TME). TAMs play a crucial dual role in either promoting or suppressing tumor development, depending on the factors that polarize them toward pro-tumorigenic or anti-tumorigenic phenotypes, as well as their interactions with cancer cells and other stromal components. In this review, we critically examine recent studies on TAMs in CCA, detailing the expression patterns and prognostic significance of different TAM subtypes in CCA, the mechanisms by which TAMs influence CCA progression and immune evasion, and the potential for reprogramming TAMs to enhance anticancer therapies. This review aims to provide a framework for deeper future research.

## 1 Introduction

CCA, the second most common primary malignancy of the liver, accounts for 10-15% of hepatic cancers (1, 2). Risk factors for CCA, which vary by location and ethnicity, mainly include hepatic fluorosis, metabolic syndromes like obesity and diabetes, non-alcoholic fatty liver disease, excessive alcohol consumption, and hepatitis B or C virus infections (3–6). CCA is traditionally classified into extrahepatic (eCCA) and intrahepatic (iCCA) forms, with eCCA further divided into distal (dCCA) and perihilar (pCCA) subtypes (1). The subtypes display distinct etiologies, prognoses, and treatment approaches. Surgery remains the cornerstone treatment for CCA (7). The anatomical location of the CCA dictates the surgical approach: hepatectomies for iCCAs and hepatopancreaticoduodenectomies for eCCAs. Although adjuvant chemotherapy post-surgery might improve survival, over half of CCA patients experience recurrence post-operation (8). The asymptomatic nature of CCA in its early stages often leads to late-stage detection, rendering many patients ineligible for surgery. For advanced stages, the leading chemotherapy regimen combines gemcitabine with cisplatin, followed by fluorouracil and oxaliplatin as secondary treatment. Nevertheless, the efficacy of these treatments in targeting CCA remains modest. Therefore, a deeper understanding of the mechanisms underlying CCA development and progression, as well as the creation of more effective treatment strategies, is crucial.

TAMs have recently emerged as a central focus of research. A substantial body of studies supports their critical role in modulating the tumor microenvironment, significantly impacting tumor progression and dissemination (9–11). TAMs originate from circulating monocytes, which are summoned to neoplastic sites by an array of chemokines and growth factors, such as CCL2 and CSF-1, released by cancer cells (12–18). Once infiltrated, these macrophages undergo a complex differentiation trajectory, steered by the local environmental cues, evolving from a classical pro-inflammatory (M1) to an alternative anti-inflammatory (M2) status (19–22). Nonetheless, within the actual tumor microenvironment, the polarization spectrum of TAMs extends beyond the simplistic M1/M2 dichotomy. The oncogenic capacities of TAMs are evident in their ability to spur angiogenesis and facilitate tissue restructuring and healing (12, 23–25). Furthermore, TAMs can suppress anti-tumor immunity by emitting a slew of cytokines and enzymes, orchestrating an immunosuppressive environment, thus enabling malignant cells to elude immune detection (26–29). Interestingly, evidence also suggests that TAMs can, under specific conditions, trigger cellular apoptosis and curtail angiogenesis, thus exerting anti-tumor effects (30, 31). The complex, dualistic nature of these roles heightens the imperative to decipher the molecular underpinnings that govern TAM recruitment, differentiation, and roles within the cancer ecosystem, as well as the therapeutic potential in targeting these entities.

This review aims to examine contemporary research on macrophages in CCA, compile the expression profiles and prognostic significance of distinct TAM subtypes within CCA, uncover the mechanisms by which TAMs influence CCA progression and immune evasion, and explore the therapeutic potential of reprogramming these TAMs to enhance anti-cancer therapies. Our review will serve as a foundation for further investigation into the complex TAM-CCA interplay.

## 2 Expression pattern and prognostic significance of TAM in CCA

### 2.1 Expression pattern

The expression pattern of TAM in the tumor microenvironment and its prognostic value have received extensive attention in recent years. The current study classifies macrophages into two main phenotypes: type M1 and type M2. M1 macrophages, also known as pro-inflammatory macrophages, have surface markers including CD80, CD86, MHC II, IL-12, and iNOS (32, 33). CD80 promotes T cell activation and anti-tumor immune response by binding to CD28 on the surface of T cells (34). Similar to CD80, CD86 is also a co-stimulatory molecule that enhances T cell activation and proliferation by binding to CD28 on T cells, aiding in the anti-tumor immune response (35). MHC II (major histocompatibility complex class II) activates CD4+ T cells by presenting antigens, thereby promoting the immune response (36). IL-12 is a cytokine secreted by macrophages and dendritic cells that promotes the activation of T cells and natural killer (NK) cells, as well as the production of IFN-γ, thereby enhancing the anti-tumor immune response (34). iNOS (inducible nitric oxide synthase) is an enzyme that catalyzes the production of nitric oxide (NO), which has the ability to kill tumor cells and microbes, thereby participating in anti-tumor and anti-infection responses (37). M2 macrophages, also known as anti-inflammatory macrophages, have surface markers including CD163, CD206, Arginase-1, IL-10, and TGF-β (32, 33). CD163 is a scavenger receptor predominantly found on the surface of macrophages. It mediates the expression of heme oxygenase-1, which has anti-inflammatory and tissue repair functions (38). CD206 is a C-type lectin receptor involved in the recognition and clearance of pathogens. It promotes anti-inflammatory and tissue repair processes by mediating endocytosis (39). Arginase-1 is an enzyme involved in the metabolism of L-arginine. By metabolizing L-arginine, it can inhibit T cell function, promote tissue repair, and support tumor growth (40). IL-10 is an anti-inflammatory cytokine secreted by various cell types, including M2 macrophages. It functions to suppress inflammatory responses, reduce the production of pro-inflammatory cytokines, and promote immune suppression and tumor progression (41). TGF-β is a multifunctional cytokine involved in cell growth, differentiation, and immune regulation. It can inhibit the function of T cells and NK cells, promote tumor cell invasion and metastasis, and regulate extracellular matrix remodeling (42).In general, a high density of M1-type macrophages is associated with a better prognosis in tumor patients. However, an increase in M2-type macrophages is often associated with a worse prognosis. It is important to note that the complexity of the tumor microenvironment means that exploring its prognostic value solely on the basis of the M1/M2 dichotomy is limited. Recent studies are attempting to achieve a more refined classification of TAMs to better reflect their functional diversity. One research team integrated data from 32 studies across 17 cancer types using single-cell RNA sequencing technology to construct a detailed macrophage diversity atlas, ultimately identifying 23 macrophage subpopulations. Cluster 1 macrophages upregulate M2 polarization-associated genes SELENOP and SLC40A1, indicating immune regulatory functions. Clusters 2 and 6 are associated with inflammatory responses, showing upregulation of genes like C3 and PLD4. Clusters 3 and 4 upregulate TREM2 and APOE, which are linked to resistance to immunotherapy. Cluster 14 shows upregulation of genes related to cell cycle and DNA replication, such as H4C3 and TOP2A, reflecting proliferative functions. Cluster 9 macrophages upregulate genes related to angiogenesis, including VEGFA, VCAN, and THBS1 (43).

Previous studies have shown that the number of macrophages in CCA tends to increase compared to adjacent non-cancerous tissue (44–46). However, due to the substantial heterogeneity of CCA, different studies have reported varying expression patterns of TAMs. Some research indicates that M0 macrophages increase significantly in iCCA, M2 macrophages decrease significantly, and M1 macrophages show no significant difference (44). Conversely, another study reported that the expression of both M1 and M2 macrophage markers was higher in iCCA compared to adjacent non-cancerous samples, with M2 macrophages predominating in iCCA tissue (45).

Many factors in the tumor microenvironment can affect the expression of TAMs. Plasma levels of IL-33, ST2, and MIF are significantly higher in iCCA patients compared to healthy controls. IL-33/ST2 signaling-related markers are also significantly increased in tumor samples. The expression of IL-33 and ST2 in iCCA is positively correlated with M1 and M2 macrophages, and is also positively correlated with the invasive clinicopathological features of CCA (45). New findings by Boulter and colleagues reveal that the expression of two ligands, Wnt7B and Wnt10A, is elevated in CCA tissues. The Wnt7B protein is present throughout the tumor stroma and co-localizes with the CD68+ macrophage subtype. During the development of CCA, the canonical Wnt signaling pathway is progressively activated. Depleting macrophages removed the primary source of Wnt7B and blocked the activation of the canonical Wnt pathway, resulting in a decrease in tumor number and size in the model (47).

In addition, factors such as the level of CD14+CD16+ monocytes, cancer stem cells (CSCs), and Tregs can also significantly affect the expression and function of TAMs (48–50). Increased levels of CD14+CD16+ monocytes are correlated with the degree of tumor-associated macrophage infiltration. These monocytes exhibit characteristics that promote tumor progression, including high expression of adhesion molecules and clearance receptors that enable them to adhere strongly to endothelial cells, while peripheral blood monocytes also show high expression of genes associated with growth and angiogenesis factors (48). The mediators released by CSCs, including IL-13, IL-34, and bone activin, can promote macrophage differentiation and invasion, contributing to tumor progression *in vivo* (49). Additionally, the number of regulatory T cells is positively correlated with the infiltration of CD68+ and CD163+ macrophages (50). One study analyzed 144,878 cells from 14 pairs of iCCA tumor and non-tumor liver tissues using single-cell RNA sequencing and found differential expression of S100P and SPP1 in iCCA portal macrotubules (iCCAphl) and peripheral microtubules (iCCApps). Compared to S100P + SPP1- iCCAphl, S100P-SPP1+ iCCApps exhibited increased SPP1+ macrophage invasion, reduced invasiveness, and improved survival (51). Furthermore, the expression of ORM2 and SPARCL1 was negatively and positively correlated with macrophage infiltration levels, respectively (52, 53).

Fatty acid biosynthesis and the expression of the key enzyme FASN were significantly increased in iCCA patients infected with Clonorchis sinensis. iCCA cell lines treated with the excreted/secreted products of Clonorchis sinensis showed elevated levels of FASN and free fatty acids. These metabolic changes are closely associated with impaired TAM and T cell function, which contribute to the formation of immunosuppressive microenvironments and tumor progression (54).

**Figure 1:**
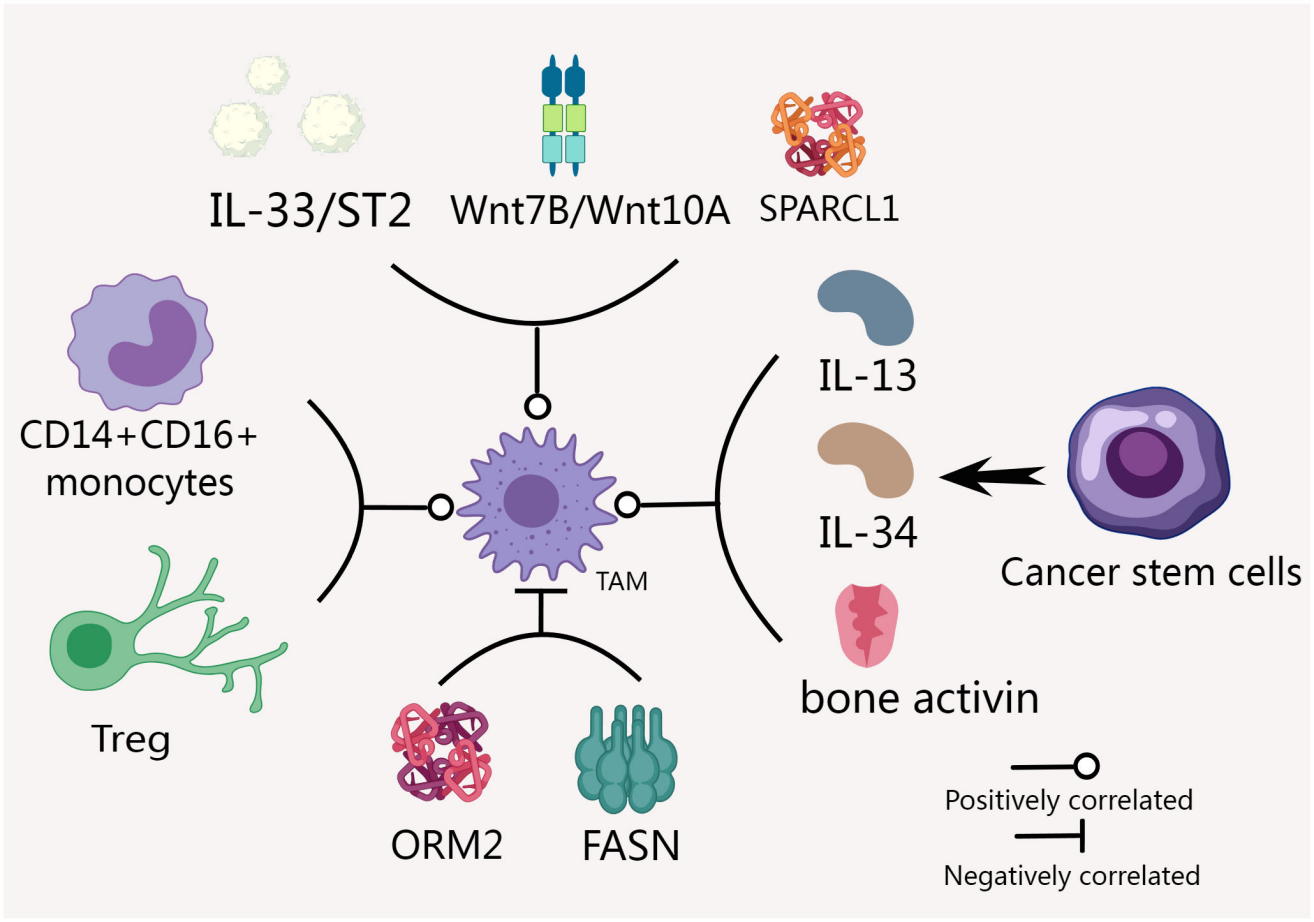
Many factors affect TAM expression in CCA. IL-33, ST2, Wnt7B and Wnt10A were positively correlated with the expression of TAM (45, 47). Factors such as CD14+CD16+ monocyte level, CSCs and Tregs can also significantly affect the expression and function of TAM (48, 49). The expression of ORM2 and SPARCL1 were negatively and positively correlated with the invasion level of TAM (52, 53). FASN and TAM function impairment are closely related (54).

We created **Figure 1** to provide a clearer visualization of the factors affecting TAM expression.

### 2.2 Prognostic significance

Previous studies on TAMs have shown that increased infiltration of M2 macrophages is associated with a poor prognosis in tumors (32, 55, 56), a finding that has also been confirmed in CCA (57).

For example, studies have shown that iCCA patients with high CD163+ macrophage counts have poor disease-free survival, but there is no significant correlation between macrophage density and overall survival (50). Another study has also shown that when the number of CD86+ TAMs is reduced and the number of CD206+ TAMs is increased, patients experience poorer prognosis and a higher likelihood of recurrence after surgery compared to patients with higher numbers of CD86+ TAMs and fewer CD206+ TAMs (58).

However, these conclusions do not appear to be absolute. Studies have shown that high-density M1-like TAMs are significantly associated with better overall survival (OS) in iCCA, whereas tumor-promoting M2-like TAMs are not associated with OS in iCCA. Conversely, in dCCA, M2 TAM density was significantly associated with poorer OS, while M1 TAM density was not associated with OS in dCCA (59). Among the different bone marrow subtypes of CCA examined, researchers found that TAM cells expressed the highest proportion of PD-L1. In both iCCA and dCCA, higher densities of PD-L1+ M1 TAMs or PD-L1+ M2 TAMs were associated with poorer survival, despite previous studies indicating that M1 TAMs are generally associated with better survival. Interestingly, when the combination of PD-L1+ M2 TAMs and PD-L1− M2 TAMs was used as a biomarker, approximately 80% of dCCA patients with low PD-L1+ M2 TAM density and high PD-L1− M2 TAM density exhibited extended survival. This finding not only affirms the role of PD-L1+ M2 TAMs in tumor promotion but also suggests that PD-L1− M2 TAMs are associated with better prognosis in dCCA and may also possess antitumor functions (59). This highlights that simple TAM polarization classifications may not be sufficient to directly predict patient prognosis, emphasizing the need to consider the type of CCA and other factors.

Further studies have shown that high activation of CD47/SIRPα and PD-1/PD-L1 signaling in CD68+ macrophages in iCCA is associated with poor prognosis (44). M-CSF-dependent peri-tumor macrophage infiltration and central-tumor macrophage infiltration independent of M-CSF were predictors of better and worse postoperative outcomes in iCCA patients, respectively. Studies have also suggested that factors in the tumor microenvironment, such as hypoxia, may influence the behavior of infiltrating macrophages in iCCA (60, 61). Other studies have found that patients with a high density of TAMs in the tumor invasive front (TIF) have a higher rate of local and distant tumor recurrence. A high density of TAMs was associated with significantly lower overall recurrence rates and improved relapse-free survival (62). Research has also shown that the neutrophil-to-lymphocyte ratio (NLR) and lymphocyte-to-macrophage ratio (LMR) are significantly associated with patient survival. High NLR and low LMR are linked to poorer clinical outcomes (63). Additionally, tumors with reduced expression of tumor-derived granulocyte macrophage colony-stimulating factor (GM-CSF) showed improved overall survival after resection (64).

## 3 Key factors regulating TAM status in CCA

### 3.1 TWEAK

Overexpression of tumor necrosis factor-like weak inducer of apoptosis (TWEAK) and its receptor fibroblast growth factor-inducible 14 (Fn14) has been detected in human CCA samples and observed in experimental animal models. Research has found that TWEAK regulates the secretion of CCA cytokines and induces the polarization of pro-inflammatory CD206+ macrophages. Drugs that block the chemokine monocyte chemoattractant protein 1 (MCP-1 or CCL2) downstream of TWEAK can significantly reduce the growth of CCA xenografts, while TWEAK overexpression promotes the proliferation and collagen deposition of cancer-associated fibroblasts. In laboratory animals, Fn14 gene knockout significantly reduced inflammation, fibrosis, and catheter-related responses. It is suggested that TWEAK/Fn14 affects tumor growth and the tumor microenvironment in CCA by regulating the polarization of macrophages and the proliferation of cancer-related fibroblasts. Therefore, targeting TWEAK/Fn14 and its downstream signaling pathways may offer a potential therapeutic strategy to inhibit CCA development and tumor growth (65).

### 3.2 SPARC

The secreted protein acidic and rich in cysteine (SPARC) is elevated in M2-polarized macrophages and TAMs. Downregulating SPARC can inhibit M2 polarization of macrophages, and this silencing effect can reduce the influence of M2 macrophages on CCA cell proliferation, migration, and angiogenesis. Additionally, knockdown of SPARC also blocks M2 polarization of macrophages by inhibiting the PI3K/AKT signaling pathway. Furthermore, activation of the PI3K signaling pathway can counteract the effects of SPARC knockdown on M2 macrophage-induced CCA cell proliferation, migration, and angiogenesis (66).

### 3.3 PCAT6

The increased expression of long non-coding RNA (lncRNA) PCAT6 in CCA patients and their macrophages suggests that it may play a key role in regulating macrophage function and promoting CCA development. Decreased PCAT6 levels can stimulate the immune response and inhibit tumor growth, while increased PCAT6 leads to the polarization of macrophages towards the M2 type, which promotes tumor growth. The study also indicates that miR-326 is a target of PCAT6, and reducing the level of PCAT6 can inhibit M2-type polarization of macrophages, while using inhibitors of miR-326 can reverse this effect. Upregulation of PCAT6 causes ROS accumulation and mitochondrial and metabolic dysfunction in macrophages, whereas the use of miR-326 mimics can counteract these effects. Additionally, RohA, as a downstream target of miR-326, plays a crucial role in this process. Therefore, the PCAT6/miR-326/RohA axis is important in regulating the immune response of CCA macrophages, suggesting that PCAT6 may be a potential target for CCA immunotherapy (67).

### 3.4 ALOX5

5-lipoxygenase (ALOX5) is a key lipid metabolic gene in iCCA that influences M2 macrophage infiltration in the TME. LTB4, a metabolite of ALOX5, activates the PI3K pathway by binding to BLT1/BLT2 on the surface of TAMs, thereby promoting the migration of M2 macrophages around the tumor and ultimately facilitating the progression of iCCA. Targeting CSF1R in combination with ALOX5 inhibitors can effectively reduce tumor volume and the extent of M2 macrophage invasion (68).

### 3.5 DKK1

Dickkopf-1 (DKK1) is associated with poor prognosis in iCCA. Overexpression of DKK1 enhances the transmission of chemokine and cytokine signals, thereby promoting the recruitment of regulatory macrophages and fostering the formation of a tolerant microenvironment containing increased regulatory T cells. Additionally, in patient tissue and gene expression data, DKK1 was found to be associated with the expression of FOXP3 and regulatory T cells. The use of DKN-01, a therapeutic drug targeting DKK1, can effectively reduce the tumor burden (69).

### 3.6 miR-451a

miR-451a has significant antitumor effects on gallbladder cancer (GBC), gemcitabine-resistant GBC (GR-GBC), and gemcitabine-resistant CCA (GR-CCA) cell lines. In both GBC and GR-GBC, miR-451a inhibits cell proliferation, induces apoptosis, and reduces chemotherapy-resistant phenotypes such as epithelial-mesenchymal transition. The main mechanism may involve the negative regulation of the phosphatidylinositol 3-kinase/AKT pathway, partly through direct downregulation of macrophage migration inhibitors (70).

### 3.7 Fucosyl-Agalactosyl IgG_1_

The proportion of serum fucosyl-agalactosyl IgG_1_ (IgG_1_-G0F) in the serum of patients with CCA is associated with poor tumor differentiation and metastasis. This type of IgG upregulates the TAM markers CD163 and CD204 in human U-937 cells and surrounding macrophages. The study also used mixed tumor cells to identify positive and negative feedback loops of transforming growth factor-β1 and interferon-γ on IgG lactose, and confirmed them in patient serum. Therefore, the presence of non-lactose IgG can activate TAMs, further promoting tumor migration and CCA recurrence (71).

### 3.8 CSEV

Chronic infection with liver flukes (such as Clonorchis sinensis) can cause severe biliary tract damage, leading to cholangitis, biliary fibrosis, and even bile duct cancer. Studies have shown that the release of extracellular vesicles by C. sinensis (CSEV) induces the activation of M1-like macrophages, leading to severe biliary tract injury. Csi-let-7a-5p was found to be enriched in CSEV. Csi-let-7a-5p promotes the activation of M1-like macrophages by targeting Socs1 and Clec7a, thus contributing to biliary tract injury. Silencing CSEV Csi-let-7a-5p tends to reduce pro-inflammatory responses and biliary tract damage, involving NF-κB signaling pathways regulated by Socs1 and Clec7a (72).

### 3.9 CSC

Cancer stem cells (CSCs) are considered potential drivers of cancer occurrence, metastasis, and recurrence, and their interactions with macrophages have been analyzed through both direct and indirect co-culture. The results showed that direct co-culture increased the proportion of the CSC population and induced the polarization of both M1 and M2 TAMs, suggesting bidirectional crosstalk between macrophages and CSCs, which promotes CSC renewal and TAM polarization (73).

We created **Table 1** to provide a more intuitive representation of the key factors regulating TAM status.

**Table 1:** Key factors regulating TAM Status in CCA.

Key factor	Status	Mechanism	Ref
TWEAK	Polarization	TWEAK regulates the secretion of CCA cytokines and induces the polarization of pro-inflammatory CD206+ macrophages	(65)
SPARC	Polarization	Silencing SPARC inhibits the PI3K/AKT signaling pathway and prevents M2 polarization of macrophages	(66)
PCAT6	Polarization	PCAT6 promotes M2-type polarization, ROS accumulation, and mitochondrial and metabolic dysfunction in macrophages via the miR-326/RohA axis	(67)
IgG_1_-G0F	Polarization	IgG_1_-G0F upregates TAM markers CD163 and CD204 and promotes M2 polarization	(71)
Csi-let-7a-5p	Polarization	Csi-let-7a-5p promotes activation of M1-like macrophages by targeting Socs1 and Clec7a	(72)
ALOX5	Infiltration	LTB4, a metabolite of ALOX5, binds to BLT1/BLT2 on the surface of TAM to activate the PI3K pathway, thereby promoting the migration of M2-type macrophages around the tumor.	(68)
DKK1	Infiltration	Overexpression of DKK1 enhances the transmission of chemokine and cytokine signals, thus promoting recruitment of macrophages	(69)
miR-451a	Infiltration	miR-451a inhibits the chemotherapy-resistant phenotype of CCA by negatively regulating the PI3K/AKT pathway and directly down-regulating macrophage migration inhibitors	(70)

## 4 Interaction between TAM and CCA

### 4.1 SHH/GLI2/TGF-β1

Under hypoxic conditions, SHH signaling pathways and endoplasmic reticulum stress (ERS) in CCA tumor tissues or tumor cell lines are activated. When tumor cells are co-cultured with macrophages, the presence of CCA cells increases the proportion of M2-polarized TAMs and the level of transforming growth factor beta 1 (TGF-β1) secreted by TAMs, while downregulation of SHH expression reverses these increases. Additionally, overexpression of GLI2 in TAMs or stimulation of TAMs increases the expression level of TGF-β1. Under co-culture conditions, interference with GLI2 expression in TAMs reduces TAM-induced biliary cancer cell migration, invasion, and endoplasmic reticulum homeostasis. In conclusion, bile duct cancer cells regulate TAM polarization and TGF-β1 secretion through the paracrine SHH signaling pathway, thereby promoting the growth, epithelial-mesenchymal transition (EMT), and endoplasmic reticulum homeostasis of bile duct cancer cells through TGF-β1 (74).

### 4.2 CD47

Studies have shown that CD47 is highly expressed in CCA compared to HCC. CD47-blocking antibodies can interfere with the interaction of CD47 with signal regulatory protein-α (SIRPα), thereby promoting macrophages to phagocytose cancer cells. Anti-CD47 treatment alleviated tumor colonization in a liver metastasis model, and dense macrophage infiltration was observed. The effectiveness of anti-CD47 highlights its ability to enhance macrophage activity. Additionally, the production of inflammatory cytokines, such as IL-6 and IL-10, was increased in macrophages exposed to CCA conditions, suggesting that CCA alters macrophages, contributing to cancer progression. Therefore, interfering with the CD47-SIRPα interaction promotes macrophage phagocytosis across all macrophage subtypes, thereby inhibiting the growth and metastasis of CCA (75).

### 4.3 aPKCγ-CCL5

M2 macrophages promote the tumor progression of CCA. Overexpression of aPKC and infiltration of M2-type macrophages are associated with metastasis and poor prognosis in CCA patients. Patients with CCA who had low M2 macrophage infiltration or low aPKC gene expression responded better to postoperative gemcitabine chemotherapy. Further studies have shown that TGF-β1 derived from M2-type macrophages induces EMT and gemcitabine resistance in CCA through the NF-κB signaling pathway mediated by aPKC-γ. Additionally, the secretion of CCL5 appears to be more abundant in CCA cells associated with aPKC-γ-mediated EMT, which can regulate the recruitment and polarization of macrophages. The simultaneous delivery of GEM and aPKC using magnetic siRNA mediated by cationic liposomes can significantly inhibit macrophage invasion and the development of CCA (76).

### 4.4 STAT3

Previous studies revealed that the supernatant of tumor cells from specific cell lines promotes the activation of signal transducer and activator of transcription 3 (STAT3), a process that drives the migration of macrophages toward the M2 phenotype. The supernatant of the HUCT1 cell line plays a particularly significant role in this process, as it not only initiates the activation of STAT3 but also stimulates the production of a series of cytokines and bioactive molecules, including IL-10, vascular endothelial growth factor A (VEGF-A), TGF-β, and matrix metalloproteinase 2 (MMP-2). Reducing STAT3 expression using siRNA technology can significantly decrease the production of IL-10 and VEGF-A. These findings support the hypothesis that tumor-associated macrophages promote cancer progression through STAT3 activation, and CD163 may be used as a potential biomarker to measure M2-type macrophages in iCCA patients and predict clinical outcomes (50).

### 4.5 IRG1

The expression of immune response gene 1 (IRG1) in M2 macrophages is low. Overexpression of IRG1 can inhibit the polarization of macrophages towards the M2 type, thus suppressing the proliferation, invasion, and migration of iCCA, while IRG1 knockdown has the opposite effect. Mechanistically, IRG1 inhibits the expression of the pro-tumor chemokine CCL18, thereby impeding the progression of iCCA by regulating STAT3 phosphorylation (77).

### 4.6 IL-10/STAT3

In a model study using experimental mice, it was observed that the number of M2-type macrophages in iCCA tissue was significantly higher than that in normal bile duct tissue, and there was a marked increase in M2-type macrophage infiltration surrounding the tumor. Experiments demonstrated that iCCA cells could induce macrophages to transform into M2-TAMs, and co-culturing these transformed macrophages with iCCA cells could accelerate the proliferation, invasion, and EMT of iCCA cells. At the molecular level, M2-TAMs induced by iCCA cells promote tumor growth and invasion through the EMT signaling pathway involving IL-10 and STAT3, suggesting a potential new target for iCCA therapy (78).

### 4.7 1,2-DCP

Based on an epidemiological study of an outbreak of CCA among offset printing workers exposed to 1,2-dichloropropane (1,2-DCP) in Japan, 1,2-DCP has recently been reclassified as a Group 1 carcinogen by IARC. A previous genome-wide mutation analysis of four CCA patients exposed to 1,2-DCP suggested that activation-induced cytidine deaminase (AID) was involved in the development of CCA, based on specific features of the mutation pattern. The administration of 1,2-DCP alone did not alter the expression of AID in bile duct cells but significantly increased the expression of the pro-inflammatory factor TNF-α in macrophages. TNF-α treatment upregulates the expression of AID in biliary duct cells, with the involvement of the NF-κB pathway. Exposure to 1,2-DCP also increased the expression of AID in bile duct cells co-cultured with macrophages, leading to enhanced DNA damage (79).

### 4.8 LPS/STAT3

Previous studies investigated the abnormal activation of the STAT protein family in human and hamster CCA tissues induced by liver fluke (Opisthorchis viverrini) infection. Activation of STAT1, STAT2, STAT3, STAT4, and STAT6 was observed in expanded bile duct epithelium and tumor cells, while expression of STAT5a and STAT5b was found in macrophages and connective tissues surrounding the tumor. In poorly differentiated tumor samples, the expression levels of STAT3 and STAT5b were significantly increased, with STAT3 expression being particularly associated with shorter patient survival. In hamster models infected with liver flukes, STATs expression exhibited a gradual upward trend as cancer progressed, especially within 30 days after infection, revealing a link between inflammation and STATs activation. Notably, LPS-induced macrophage-conditioned media promoted the activation of STAT3 in CCA cells (80).

We created **Table 2** to illustrate the interaction between TAMs and CCA more clearly.

**Table 2:** Interaction between TAM and CCA.

Key factor	Type	Mechanism	Ref
SHH	M2	CCA cells regulate TAM M2 polarization and TGF-β1 secretion through paracrine SHH signaling pathway, and then promote biliary cancer cell growth, EMT and endoplasmic reticulum homeostasis through TGF-β1.	(74)
CD47/SIRRP-α	TAM	Interfering with the CD47-SIRRP-α interaction promotes phagocytosis of all macrophage subtypes, thereby inhibiting the growth and metastasis of CCA.	(75)
aPKC-γ/CCL5	M2	TGF-β1 in M2 macrophages induces EMT and gemcitabine resistance in CCA through the APKC-γ-mediated NF-κB signaling pathway. At this time, the secretion of CCL5 in CCA cells increases, thereby regulating the recruitment and polarization of macrophages	(76)
STAT3	M2	M2-type macrophages stimulate the secretion of IL-10, VEGF-A, TGF-β and MMP-2 through STAT3 activation, and promote CCA	(50)
IRG1	M2	Overexpression of IRG1 can inhibit the polarization of macrophages to M2 type and inhibit the expression of CCL18, and then regulate the phosphorylation of STAT3 to inhibit the progression of CCA.	(77)
IL-10/STAT3	M2	M2-TAM induced by CCA cells promotes tumor growth and invasion through EMT signaling pathways of IL-10 and STAT3	(78)
1,2-DCP	TAM	1,2-DCP significantly increased the expression of TNF-α in macrophages, and further increased the expression of AID in bile duct cells through the NF-κB pathway	(79)

**Figure 2:**
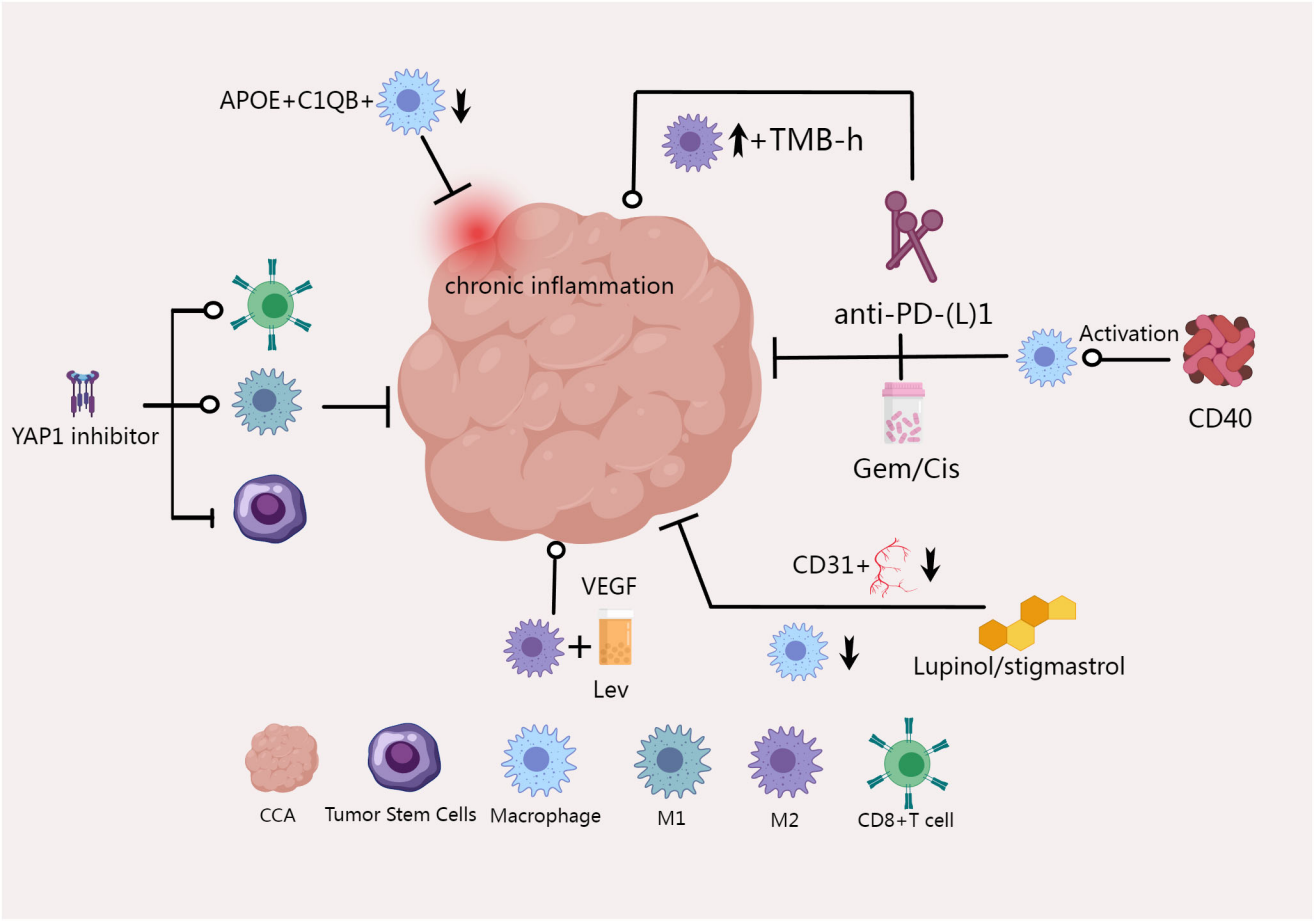
CCA can regulate TAM polarization and TGF-β1 secretion through paracrine SHH signaling pathway, thus promoting the growth of bile duct cancer cells (74); TWEAK and its receptor Fn14 are overexpressed in CCA, inducing the polarization of pro-inflammatory CD206+ macrophages, and drugs that block MCP-1 or CCL2 can significantly inhibit CCA (65); TGF-β1 derived from M2-type macrophages induces CCA EMT and gemcitabine resistance through aPKC-γ-mediated NF-κB signaling pathway (76); Knocking down SPARC can block M2 polarization of macrophages by inhibiting PI3K/AKT signaling pathway (66); miR-451a can also inhibit PI3K/AKT pathway and regulate macrophages (70); M2-TAM induced by iCCA cells promotes tumor growth and invasion through EMT signaling pathways of IL-10 and STAT3 (78); IRG1 overexpression also inhibited macrophage polarization toward M2 type (77); LTB4, a metabolite of ALOX5, activates the PI3K pathway by binding to BLT1/BLT2 on the surface of TAM, thereby promoting the migration of M2 macrophages around the tumor and ultimately promoting the progression of iCCA (68).

In the tumor microenvironment, TAMs exert their function through a series of complex cascade reactions. Numerous studies are devoted to understanding the regulatory mechanisms of TAMs and how they affect and promote tumor progression through multiple pathways. We created **Figure 2** to illustrate this complex mechanism more clearly.

## 5 Therapeutic potential of TAM in CCA

Targeting TAMs for cancer treatment or using TAMs to predict the efficacy of tumor therapies is currently a popular research direction. Existing clinical trials, including NCT00992186, NCT02996110, NCT02584647, NCT02452424, and NCT03101254, have preliminarily demonstrated the effectiveness of targeting TAMs in various cancers such as prostate cancer, kidney cancer, melanoma, and breast cancer (81). However, there are currently no clinical trials targeting TAMs in CCA. Conventional treatments for CCA mainly include surgical resection, chemotherapy, and radiotherapy. As an emerging therapeutic approach, immunotherapy is becoming increasingly important. To improve the effectiveness of CCA treatment, many strategies are focusing on targeted therapies for macrophages. The goal of these strategies is to alter the behavior of macrophages to either promote tumor inhibition or enhance their ability to inhibit tumors, thereby modulating the tumor microenvironment and ultimately improving therapeutic outcomes.

In the course of chemotherapy and immunotherapy for CCA, macrophages can play a crucial role in therapeutic efficacy. Studies have shown that in CCA patients with high tumor mutation burden (TMB-H), depletion of T cells and increased counts of M2 macrophages are observed in the anti-PD-(L)1 non-responsive group (82). Additionally, the use of CD40 agonists to stimulate antigen-presenting cells (including macrophages and dendritic cells) in combination with anti-PD1 therapy can significantly reduce tumor burden. This combination therapy increases the number and activation of CD4+ and CD8+ T cells, natural killer cells, and myeloid cells in both tumor and non-tumor tissues. Depleting macrophages, dendritic cells, CD4+ T cells, or CD8+ T cells nullifies the effectiveness of the treatment. The combination of anti-CD40/PD-1 therapy and chemotherapy (gemcitabine/cisplatin) can significantly improve patient survival. It is suggested that CD40-mediated activation of macrophages and dendritic cells can significantly enhance the response of CCA to PD-1 therapy (83). Macrophage capping protein (CapG) has also been shown to be associated with the response of CCA to gemcitabine therapy. The expression of CapG is related to lymphatic infiltration and overall survival, and is an independent prognostic factor affecting survival. Therefore, CapG is considered a novel candidate biomarker for predicting response to and survival with gemcitabine therapy in patients with CCA (84). When co-cultured with M2 macrophages, the apoptosis of bile duct cancer cells induced by Lenvatinib was significantly reduced, and angiogenesis-related factors were significantly increased. Conversely, when co-cultured with M1 macrophages, Lenvatinib increased the apoptosis of bile duct cancer cells. This suggests that compared to M1 macrophages, the antitumor effect of Lenvatinib on CCA is inhibited by M2 macrophages through immunomodulatory regulation, which may be related to tumor angiogenesis factors in M2 macrophages (85).

The Hippo/YES-associated protein (YAP) pathway impacts all stages of tumorigenesis, and high expression of YAP1 is inversely correlated with survival in CCA patients. The YAP1 pathway inhibitor verteporfin reduced tumor burden in mice and increased the proportion of TAM M1 cells, while also enhancing the percentage of activated CD8+ T cells and decreasing the proportion of stem-like malignant cells. It is suggested that verteporfin may inhibit tumorigenesis by polarizing anti-tumor TAMs, activating CD8+ T cells, and reducing the proportion of stem-like cells in the tumor microenvironment (86). Lupinol and stigmasterol, the main phytosterols found in a variety of herbs, have anti-inflammatory activity and are considered candidates for anti-cancer drugs. The use of lupinol and stigmasterol in CCA can disrupt tumor angiogenesis, reduce the growth of xenografts of CCA tumors, and lower the CD31-positive blood vessel content and macrophage recruitment after treatment. These results suggest that they are promising candidates for anticancer treatment of CCA tumors (87). Recently, a new APOE+C1QB+ macrophage subtype has been identified that can reshape chronic inflammatory subtypes and negatively impact the prognosis of iCCA. Targeting APOE+C1QB+ TAMs is a potential immunotherapeutic strategy for iCCA (88). Targeted therapies against macrophages are emerging as a new frontier in the fight against CCA, and further developments in these therapies are anticipated as research progresses.

**Figure 3:**
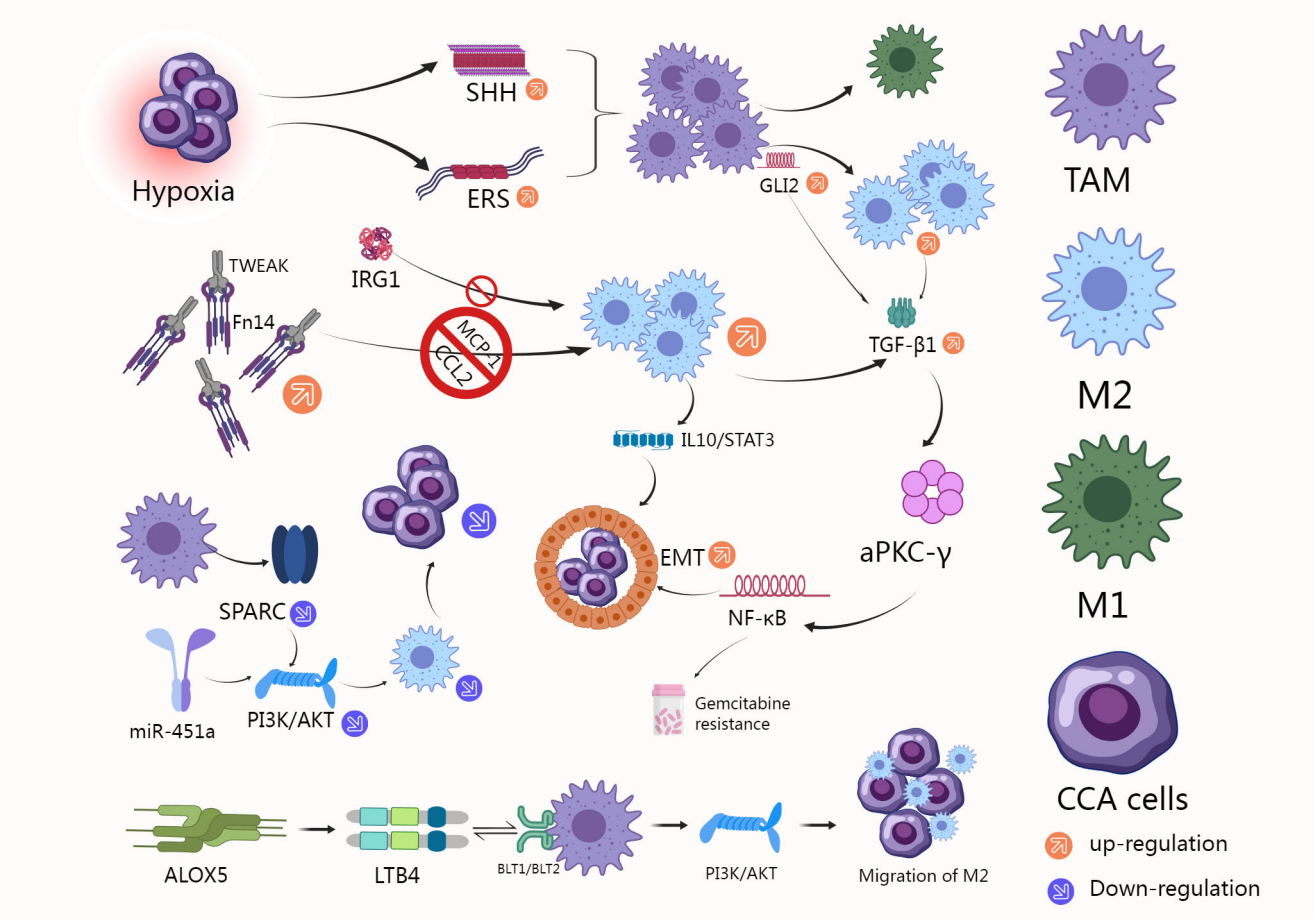
M2 macrophage count is increased in tumors of TMB-h CCA patients who do not respond to anti-PD-(L)1, and CD40-mediated macrophage activation can significantly enhance CCA response to PD-1 therapy (82, 83). M2 macrophages reduce the effect of Lenvatinib by regulating tumor angiogenesis factors (85). YAP1 pathway inhibitors induced TAM M1 polarization, increased the percentage of activated CD8+ T cell population, and decreased the proportion of stem-like cells in malignant cells to reduce tumorigenesis (86). Lucidol and stistastrol can disrupt CCA angiogenesis, reduce CD31-positive blood vessel content and macrophage recruitment (87).

We created **Figure 3** to present the existing results more intuitively.

## 6 Discussion

CCA is a malignant tumor originating from the epithelial cells of the bile ducts. CCA is characterized by high aggressiveness and poor prognosis, with early diagnosis being challenging and treatment options for advanced stages being limited. In recent years, advances in understanding the biological characteristics of CCA, particularly its immunogenicity, have opened up new avenues for treatment (89, 90). CCA cells express various tumor-associated antigens (TAAs) that can be recognized by the immune system. For example, antigens such as CEA, MUC1, and EpCAM are highly expressed in CCA and serve as potential targets for immunotherapy (91–93). Despite the typically immunosuppressive microenvironment of CCA, studies have found that some CCA tumors contain a significant number of tumor-infiltrating lymphocytes (TILs), particularly CD8+ cytotoxic T cells. These T cells have the ability to recognize and kill tumor cells, but their function is often suppressed (94). For example, Tregs and MDSCs are abundant in the CCA microenvironment. These immunosuppressive cells inhibit the function of effector T cells, facilitating tumor immune evasion (95, 96). Similar to other cancers, TAMs play a crucial role in CCA, with most TAMs exhibiting an M2 phenotype that promotes tumor progression and immune evasion. However, CCA generally has lower immunogenicity compared to highly immunogenic tumors like melanoma. Melanoma typically features a high mutation burden and diverse tumor antigens, which contribute to its elevated immunogenicity (97, 98). In contrast, CCA has a relatively low mutation burden and less diversity in tumor antigens compared to melanoma. Melanoma is characterized by a high presence of TILs, particularly CD8+ T cells, which often exhibit strong cytotoxic activity (99). In CCA, although TILs are present, their number and function are often limited, and the proportion of immunosuppressive cells is relatively high (95, 96). Although CCA also exhibits high levels of immune checkpoint molecules, the response rate to immune checkpoint inhibitors (ICIs) in melanoma is significantly higher than in CCA. This difference may be attributed to melanoma’s higher mutation burden and greater immunogenicity (100–102). In the melanoma microenvironment, although immunosuppressive cells and factors are present, the immune-activating signals are stronger, leading to a more active immune response. In contrast, the CCA microenvironment is dominated by immunosuppressive factors, making immune evasion more pronounced (103–106). Therefore, research on TAMs in CCA remains behind that of highly immunogenic tumors like melanoma. However, the advanced findings from melanoma studies can provide valuable insights and serve as a reference for TAM research in CCA.

Various factors can influence the polarization state of TAMs in CCA. For example, targets such as TWEAK, SPARC, PCAT6, and ALOX5 can affect TAM polarization characteristics in CCA. In highly immunogenic tumors like melanoma, interactions between immune cells also play a crucial role in TAM polarization. For instance, Tregs and MDSCs can promote TAM polarization towards the M2 phenotype by secreting immunosuppressive factors like IL-10 and TGF-β, thereby supporting tumor growth and immune evasion (107–109). CD8+ T cells and NK cells can induce TAM polarization towards the M1 phenotype by secreting pro-inflammatory factors such as IFN-γ, thereby enhancing anti-tumor immune responses. Additionally, Th1 cells can secrete IFN-γ to promote TAM polarization towards the M1 phenotype (110–112). Th2 cells can secrete IL-4 and IL-13, which promote TAM polarization towards the M2 phenotype (113, 114). Th17 cells secrete IL-17, which primarily affects neutrophils but can also indirectly influence TAM polarization (115, 116). The role of B cells is more complex, with various B cell subsets influencing TAM polarization in diverse ways through the secretion of different cytokines. For example, B cells secreting IL-10 can promote TAM polarization towards the M2 phenotype, while certain pro-inflammatory B cell subsets may secrete TNF-α and other factors to support M1 polarization (117–119). In CCA, although there are a few studies exploring the impact of immune cell interactions on TAM polarization, they mostly focus on T cells, such as Tregs and CD8+ T cells. Additionally, research has increasingly investigated the heterogeneity of TAMs in tumors, with specific subpopulations identified—up to 23 distinct subgroups (43). This highlights the importance of exploring the heterogeneity of TAMs in CCA at the single-cell level, rather than relying solely on the M1/M2 classification.

In tumors, TAMs can influence tumor progression through various mechanisms. Previous studies have shown that TAMs can promote tumor growth and metastasis by directly secreting cytokines, growth factors, and chemokines. These secretions contribute to processes such as tumor angiogenesis and EMT (17, 120). Additionally, TAMs secrete immunosuppressive factors such as IL-10 and TGF-β, which inhibit the activity of effector T cells and natural killer (NK) cells, thereby promoting tumor immune evasion (120, 121). TAMs can also express PD-L1, which binds to PD-1 on T cells, thereby inhibiting T cell activity and facilitating tumor immune evasion (27). Tumor metabolism is a currently popular research area, and TAMs can influence tumor progression by modulating metabolic pathways within the tumor microenvironment. They promote the accumulation of lactate and other metabolic byproducts, which can further inhibit immune cell function. Additionally, TAMs secrete matrix metalloproteinases (MMPs) and other proteases that remodel the extracellular matrix, altering the physical and biochemical properties of the tumor microenvironment and promoting tumor progression (122). In CCA, TAMs can promote tumor progression through various pathways, such as the SHH/GLI2/TGF-β1 pathway and the aPKCγ/CCL5 pathway, as previously summarized. The identification of these key targets and pathways provides a foundation for further clinical translation.

With the advancement in TAM research, clinical trials targeting TAMs have been initiated across various cancer types. For example, CSF-1R inhibitors can reduce the number and function of TAMs by inhibiting the CSF-1R signaling pathway (123). Additionally, CCR2 antagonists can reduce TAM tumor infiltration by blocking the macrophage chemokine receptor (124). Additionally, clinical trials combining TAM-targeting therapies with immune checkpoint inhibitors are actively underway (125). Although functional targets related to TAMs in CCA are continually being identified, no clinical trials specifically targeting TAMs in CCA have been conducted so far. This area of clinical translation is likely to be a major focus for future research.

In summary, our review paints a multifaceted picture of TAMs in the context of CCA, highlighting their complex roles in the tumor microenvironment. Our comprehensive review of the existing literature indicates that TAMs contribute to the carcinogenic landscape of CCA by promoting immune suppression, angiogenesis, and tumor growth. Thus, targeting TAMs represents a potent intervention point for therapy. The potential to manipulate TAM activity and polarization offers an exciting prospect for innovative treatment strategies, which could synergize effectively with existing therapies, such as PD-1/PD-L1 blockade. Further research into TAM-mediated mechanisms of CCA tumorigenesis and immune regulation will enhance our understanding of the immunopathology of CCA and lead to the development of more effective treatment strategies.

## Glossary

CCA: Cholangiocarcinoma
TAMs: Tumor-associated macrophages
TME: Tumor microenvironment
eCCA: Extrahepatic cholangiocarcinoma
iCCA: Intrahepatic cholangiocarcinoma
dCCA: Distal cholangiocarcinoma
pCCA: Perihilar cholangiocarcinoma
CSCs: Cancer stem cells
OS: Overall survival
TIF: Tumor invasive front
NLR: Neutrophil to lymphocyte ratio
LMR: Lymphocyte to macrophage ratio
GM-CSF: Granulocyte macrophage colony-stimulating factor
TWEAK: Tumor necrosis factor-like weak inducer of apoptosis
Fn14: Factor-inducible 14
MCP-1: Monocyte chemoattractant protein 1
HCC: Hepatocellular carcinoma
SIRPα: Signal regulatory protein-α
SPARC: Secreted protein acidic and rich in cysteine
lncRNA: Long non-coding RNA
ALOX5: 5-lipoxygenase
DKK1: Dickkopf-1
STAT3: Signal transduction and activator of transcription 3
VEGF-A: Vascular endothelial growth factor A
TGF-β1: Transforming growth factor beta 1
MMP-2: Matrix metalloproteinase 2
IRG1: Immune response gene 1
MARCKS: Myristoylated rich C-kinase substrates
LPS: Lipopolysaccharide
GBC: Gallbladder cancer
GR-GBC: Gemcitabine-resistant GBC
GR-CCA: Gemcitabine-resistant CCA
1: 2-DCP1, 2-dichloropropane
AID: Activation-induced cytidine deaminase
IgG_1_-G0FFucosyl-agalactosyl: IgG_1_(IgG_1_-G0F)
CSEV: Extracellular vesicles by C. sinensis
TMB-H: High TMB
YAP: Yes associated protein
MerTK: Macrophage c-mer tyrosine kinase
TILs: Tumor-infiltrating lymphocytes
NK: Natural killer
TAA: Tumor-associated antigens
ICIs: immune checkpoint inhibitors
